# Three-Dimensional Printed Shape Memory Gels Based on a Structured Disperse System with Hydrophobic Cellulose Nanofibers

**DOI:** 10.3390/polym15173547

**Published:** 2023-08-26

**Authors:** Angelina P. Prosvirnina, Alexander N. Bugrov, Natalya V. Bobrova, Eugene V. Sivtsov, Alexandra L. Nikolaeva, Almaz M. Kamalov, Maria P. Sokolova, Michael A. Smirnov

**Affiliations:** 1 Institute of Macromolecular Compounds, Russian Academy of Sciences, Bolshoy Pr. 31, Saint Petersburg 199004, Russia; 2 Department of Physical Chemistry, Saint Petersburg Electrotechnical University (ETU “LETI”), ul. Professora Popova 5, Saint Petersburg 197022, Russia; 3 Saint Petersburg State Institute of Technology, Moskovsky Pr. 24-26/49, Saint Petersburg 190013, Russia

**Keywords:** bacterial cellulose, 3D printing, rheological properties, mechanical characteristics, shape memory effect

## Abstract

Inks for 3D printing were prepared by dispersing bacterial cellulose nanofibers (CNF) functionalized with methacrylate groups in a polymerizable deep eutectic solvent (DES) based on choline chloride and acrylic acid with water as a cosolvent. After 3D printing and UV-curing, the double-network composite gel consisting of chemically and physically crosslinked structures composed from sub-networks of modified CNF and polymerized DES, respectively, was formed. The rheological properties of inks, as well as mechanical and shape memory properties of the 3D-printed gels, were investigated in dynamic and static modes. It was shown that the optimal amount of water allows improvement of the mechanical properties of the composite gel due to the formation of closer contacts between the modified CNF. The addition of 12 wt% water results in an increase in strength and ultimate elongation to 11.9 MPa and 300%, respectively, in comparison with 5.5 MPa and 100% for an anhydrous system. At the same time, the best shape memory properties were found for an anhydrous system: shape fixation and recovery coefficients were 80.0 and 95.8%, respectively.

## 1. Introduction

Polymer gels consisting of crosslinked macromolecules (polymer network) are prospective functional materials that are intensively studied as superabsorbents [1,2,3], mechanical dampers [4,5,6], artificial implants [7,8,9,10], scaffolds for tissue engineering [11,12,13,14,15], and construction materials for soft robotics [16,17,18]. However, the strength of polyacrylate hydrogels is rather low, typically in the range 1–100 kPa [19,20,21]. This is not enough for high-end practical applications, such as artificial cartilage, where the material is required to withstand loads of 3–17 MPa [11,22]. Thus, the methods for preparation of reinforced polymer gels are intensively elaborated [23,24]. Among different methods of the reinforcement of polymer gels that can be provided by the alteration of their structure, the following can be emphasized: (i) Addition of nanoparticles with high mechanical properties, such as graphene oxide [25,26,27], cellulose nanofibers (CNF) [28,29,30], or nanocrystals [31,32]; (ii) interaction of polyampholyte polymer gels with polyvalent metal salt solutions [24,33]; (iii) formation of a double-network composed of two interpenetrating crosslinked macromolecular networks with mechanical properties significantly higher in comparison with both individual frameworks [34,35,36,37]; (iv) preparation of gels by the impregnation of a continuous reinforcing frame with monomer or prepolymer and subsequent polymerization. Examples of the latter case include acrylamide-acrylic acid copolymer [38] or gelatin crosslinked with 1-(3-dimethylaminopropyl)-3-ethylcarbodiimide hydrochloride in the framework of a bacterial cellulose hydrogel [37] that allow an increase in the strength of the gels by up to 76 and 5 MPa, respectively. However, this approach can be utilized only for the preparation of blocks and is not suitable for 3D printing. In the case of the formation of 3D printed bulk reinforced hydrogels, the more complicated methods, such as melt electrospinning writing of a polycaprolactone scaffold embedded into the gelatin methacrylamide hydrogels, were reported [39]. Using a conventional direct ink writing, the bulk reinforcement frame should be formed after the formation of a 3D printed model. This approach was demonstrated by Gao et al. [40], who fabricated a printable hydrogel for tissue engineering based on methacrylated gelatin (GelMA) with different contents of poly(N-acryloyl-2-glycine). This hydrogel was obtained using UV-induced radical polymerization, and its compressive strength was superior to that of the pristine GelMA hydrogel (12.4 MPa vs. 110 kPa). Additionally, the stiffness of a system with maximum filler content was 1.1 MPa against 158 kPa for the pure GelMA hydrogel. A similar strategy, but with nanofiller, was used in [41], where the vinyl groups of acrylic acid were a part of the ionic gel, and 3-(trimethoxysilyl)propyl methacrylate, covalently attached to the CNF surface, was crosslinked by UV irradiation after 3D printing of the models. As a result, a reinforcing frame of covalently connected CNF was formed. The crystallization of polyvinyl alcohol during the freeze–thaw procedure was used for the formation of the reinforcing frame after the polymerization of a deep eutectic solvent based on acrylic acid and choline chloride [42]. 

A further increase in the mechanical properties of the reinforcing network formed after 3D printing is anticipated to be achieved by strengthening the interactions between the modified CNF. This may be attained by changing the stabilization ability of the dispersion media. The coagulation structures or structured disperse systems can be assembled by governing the interactions between the filler particles, which form spatial cells in the bulk of the dispersion [43]. A characteristic feature of the coagulation structures, along with their relatively low strength before crosslinking, is the ability to spontaneously recover after mechanical stress, i.e., thixotropy [44]. In the work [45], the authors suggested that van der Waals, dipole–dipole interactions and hydrogen bond formation can govern the formation of the coagulation structure. In this work, we proposed the hypothesis that coagulation structures increase the strength of the crosslinked network in the reinforcing frame of polymer gels due to the formation of closer contacts between the filler particles before crosslinking. Then, crosslinking after 3D printing fixes the structure and provides a bulk reinforcement frame. This hypothesis was tested on the basis of a system consisting of CNF hydrophobized with a polysiloxane shell with methacrylate groups dispersed in a deep eutectic solvent (DES) containing acrylic acid and choline chloride, with water being used as a coagulant for the CNF. The material, consisting of a crosslinked CNF-reinforcing filler embedded in choline chloride/acrylic acid DES after UV curing, can be considered to be a double network (DN) that is built of chemically crosslinked (CNF) and physically crosslinked (polymerized DES—pDES) grids. It can be supposed that, in this system, the mechanically extended shape above the glass transition temperature of pDES (*T_g_*) can be fixed by cooling the material below *T_g_*. In a frozen state, the mechanical tension is stored in the chemically crosslinked CNF framework, which allows the sample to recover its shape when it is heated above the pDES relaxation transition temperature. Thus, manifestations of the thermally induced shape memory effect can be expected for the systems under study. The objectives of this study were the verifications of two hypotheses, namely: (1) fixing of a coagulation structure based on hydrophobized CNF with UV-curing of crosslinkable groups will allow us to push up the mechanical properties of polymer gel; and (2) a system containing a chemically crosslinked CNF network and a physically crosslinked pDES network will demonstrate shape memory behavior. To the best of our knowledge, the shape memory properties in CNF-based composite gels where *T_g_* of pDES acts as the switching temperature have not yet been reported. Also, utilizing coagulation structures for improving mechanical properties of 3D printed objects has not been not studied.

## 2. Materials and Methods

### 2.1. Materials

Lyophilized culture of Komagataeibacter xylinus was purchased from the All-Russian collection of industrial microorganisms (National Bioresource Center, Gos-NIIgenetika, Moscow, Russia) and cultivated as described earlier [41]. Peptone, yeast extract (Russian Research Center for Pharmacotherapy, Saint Petersburg, Russia), D-mannitol (LenReactiv, Saint Petersburg, Russia, CAS 69–65-8, purity 99%), urea (LenReactiv, Saint Petersburg, Russia, CAS 57–13-6, purity 98%), 3-(trimethoxysilyl)propyl methacrylate (TMSPM) (Sigma-Aldrich, Darmstadt, Germany, CAS 2530–85-0, purity 98%), ammonia (Vekton, Saint Petersburg, Russia, CAS 7664–41-7, 25% aqueous solution, analytical grade), tetrahydrofuran (THF) (Vekton, Saint Petersburg, Russia, CAS 109–99-9, reagent grade), acrylic acid (AA) (Sigma-Aldrich, Praha, Czech Republic, CAS 79–10-7, purity 99%), and 2-hydroxy-2-methylpropiophenone (Sigma-Aldrich, Milano, Italy, CAS 7473–98-5, purity 97%) were used as received. Choline chloride (ChCl) (Glentham Life Sciences Ltd., Corsham, UK, CAS 67–48-1, purity 99%) was dried under vacuum at 60 °C for at least 24 h before use.

### 2.2. Methods

#### 2.2.1. CNF Modification and Ink Preparation

Synthesis and surface functionalization of bacterial cellulose nanofibers with TMSPM were performed using the protocol given in the previous work [41]. As a result, the polysiloxane shell was formed on the surface of the CNF. After washing, the modified CNF (M-CNF) was obtained as a dispersion in THF. The compositions for 3D printing were prepared as follows. DES, consisting of ChCl (12.54 g) and AA (19.40 g) in molar ratio 1:3, was formed at 65 °C. M-CNF dispersions in THF were cooled down to −20 °C and then added to the DES in order to provide 4 wt% of M-CNF after complete THF removal and addition of water. The THF was removed by evaporation during heating of the mixture up to 70 °C with stirring until the constant mass was reached. After that, 5, 12, or 30 wt% of water was added to the ink in order to obtain compositions for 3D printing that will be denoted as M-CNF-5, M-CNF-12, and M-CNF-30, respectively. The mass of the added DES was reduced by the amount of added water in order to maintain the constant content of M-CNF in dispersion. The ink without addition of water (M-CNF-0) was also studied. The typical mass ratios of M-CNF/DES/H_2_O in the prepared inks were 0.32/7.68/0, 0.32/7.28/0.4, 0.32/6.72/0.96, and 0.32/5.28/2.4 for M-CNF-0, M-CNF-5, M-CNF-12, and M-CNF-30, respectively. Finally, 2-hydroxy-2-methylpropiophenone (2 wt% relative to AA) was added as an initiator.

#### 2.2.2. Rheology

The rheological properties of the prepared dispersions were measured using a rheometer PHYSICA MCR302 (Anton Paar, GmbH, Graz, Austria) equipped with a CP25–2 cone and plane cell (diameter of 25 mm, a cone angle of 2°, and a gap of 0.5 mm) at a constant temperature of 25 °C. Shear stress measurements were carried out at an increasing shear rate in the range from 10^−1^ to 10 s^−1^. The angular frequency sweep tests were performed at the frequency range of 0.1 to 100 rad⋅s^−1^ at a strain amplitude value within the (LVE) region: 0.1%, 0.04%, 0.05%, and 0.03% for M-CNF-0, M-CNF-5, M-CNF-12, and M-CNF-30, respectively (Figure S1).

#### 2.2.3. Atomic Force Microscopy (AFM)

A detailed investigation of the surface of M-CNF-based ionic gels with various water content was performed using a scanning probe microscope (SPM-9700HT, Shimadzu, Kyoto, Japan) operating in the tapping mode, with an NSG30-SS silicon tip (tip curvature radius 2 nm). A total of 512 × 512 points images were obtained.

#### 2.2.4. Three-Dimensional Printing

The formation of the samples for mechanical investigation based on M-CNF inks was performed on a 3D printer 3D BioScaffolder BS3.2 (GeSIM, Radeberg, Saxony, Germany) equipped with a pneumatic syringe (nozzle diameter 0.58 mm) applying a pressure 50–110 kPa. The polymerization process (UV-curing) was induced with a UV-lamp OmniCure S1500 (Lumen Dynamics, Mississauga, Ontario, Canada) operating at a power of 0.3 W cm^−2^. Curing was conducted with seven pulses with a duration of 30 s and with 30 s pauses between them.

#### 2.2.5. Fourier Transform Infrared Spectroscopy

The infrared spectra of the M-CNF-12 dispersion in DES and polymerized DES were obtained using an IRAffinity-1S spectrometer (Shimadzu, Japan). The samples were spread between thallium bromo-iodide crystal plates (KRS-5) and studied by Fourier transform infrared (FTIR) spectroscopy in the transmittance mode. Each spectrum was recorded in the range from 4000 to 400 cm^−1^ with a resolution of 2 cm^−1^ and with a total of 100 scans. 

#### 2.2.6. Mechanical Properties 

The mechanical properties were investigated using an AG-100kNX Plus (Shimadzu, Japan) setup in uniaxial stretching mode for samples obtained in the form of filaments (D = 0.8–1 mm, H = 20 mm) by using 3D printing. The traverse movement speed was 50 mm/min with a working length of 20 mm. During the experiment, the following characteristics of materials were determined: Young’s modulus (*E*), strength at break (*σ_f_*), and deformation at break (*ε_f_*).

#### 2.2.7. Dynamic Mechanical Analysis (DMA)

The temperature dependencies of the dynamic mechanical characteristics, namely the elastic modulus (*E*’) and the tangent of the angle of mechanical losses (tan *δ*), of the samples were measured on DMA 242 C equipment (NETZSCH, Selb, Germany). The measurements were performed at a frequency of 1 Hz, the rate of temperature rise was 5 °C/min, and the deformation amplitude was 0.1%. The base length of the sample was 9–10 mm. The glass transition temperature of each sample was determined as the temperature of maximum on the tan *δ*(*T*) curve. The same filaments that were used for the mechanical measurements were taken.

#### 2.2.8. Shape Memory Properties

The coefficients of shape fixation (*R_f_*) and restoration (*R_r_*) were determined from the DMA diagrams, retrieved using a DMA 242 C device (NETZSCH, Selb, Germany) with a static force (FS) 0.6 H in the temperature range from −50 °C to 30 °C. The value of *R_f_*, which determines the ability of a material to withstand the applied mechanical deformation, was calculated with the equation:

*R_f_* = (*ε*/*ε_load_*) × 100%, (1)

where *ε_load_* is the maximum deformation of a sample when cooled below the glass transition temperature in the loaded state, and *ε* is the fixed deformation after cooling and removing the load from the sample. The *R_r_*, characterizing the degree of restoration of the material to its original shape after deformation, was calculated using the ratio:

*R_r_* = ((*ε* − *ε_rec_*)/*ε*) × 100%, (2)

where *ε_rec_* is the deformation after reheating without load and restoring the shape of the sample. 

**Figure 1:**
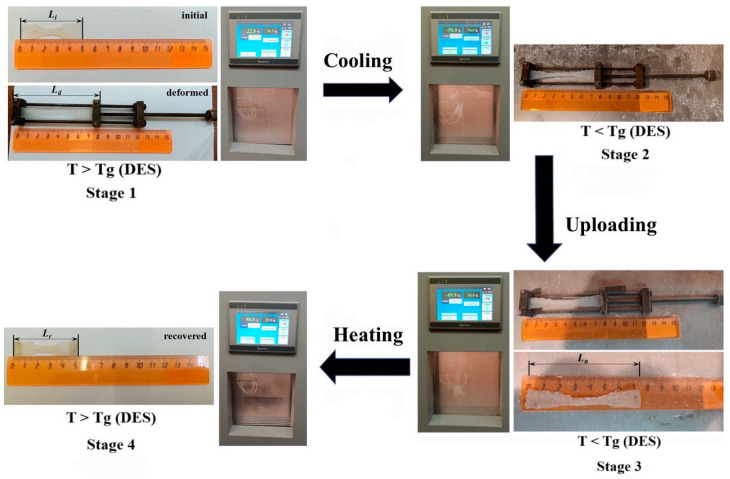
Scheme of cyclic thermomechanical tests in a static mode to determine the properties of shape memory on the example of the 3D printed model in the shape of a dog bone, obtained from M-CNF-0 dispersion.

The properties of the shape memory in the static mode were determined using cyclic thermomechanical tests. To perform this, the initial length of the 3D printed model in the shape of a dog bone was measured, after which the material was stretched by 60% at room temperature, being in a highly elastic state with the help of a device for the uniaxial orientation of the films (stage 1, Figure 1). Then, the deformed model was placed in a climate chamber and cooled to a temperature of −50 °C (stage 2, Figure 1). The sample was kept at a given temperature for 10 min to reach an equilibrium state. The load was then removed from the sample and its length was measured every 5 min until it stopped changing over time (stage 3, Figure 1). At the final stage, the deformed sample was heated to 50 °C in a climate chamber, and the final length of the model was obtained (stage 4, Figure 1).

In accordance with [46], the shape fixation coefficient for the static mode (*R_fs_*) was calculated with the following equation:

(3) $$R_{fs}=\frac{L_{u}-L_{i}\;}{L_{d}-L_{i}}\times 100\%,$$

where *L_i_* is the initial sample length, *L_d_* is the deformed sample length with loading at a temperature above the *T_g_* of DES, and *L_u_* is the equilibrium length of the deformed sample after unloading at −50 °C.

The value of shape recovery for the static mode (*R_rs_*) was calculated as:

(4) $$R_{rs}=\frac{L_{u}-L_{r}\;}{L_{u}-L_{i}}\times 100\%,$$

where *L_r_* is the unloaded sample length after heating it above the *T_g_* of DES.

## 3. Results and Discussion

### 3.1. Preparation of Dispersions

During preliminary study, the effect of water on the stability of the M-CNF dispersion in DES against delamination was investigated. For this purpose, various amounts of water were added to the M-CNF dispersion in DES, and the dispersion stability was visually determined. It was found that dispersions with water contents higher than 30 wt% are unstable under mechanical load, while concentrations of water >50 wt% cause delamination at rest. To investigate the influence of the gradual increase in water content below and close to the instability threshold, dispersions containing 5, 12, and 30 wt% were selected for further work.

### 3.2. Atomic Force Microscopy

**Figure 2:**
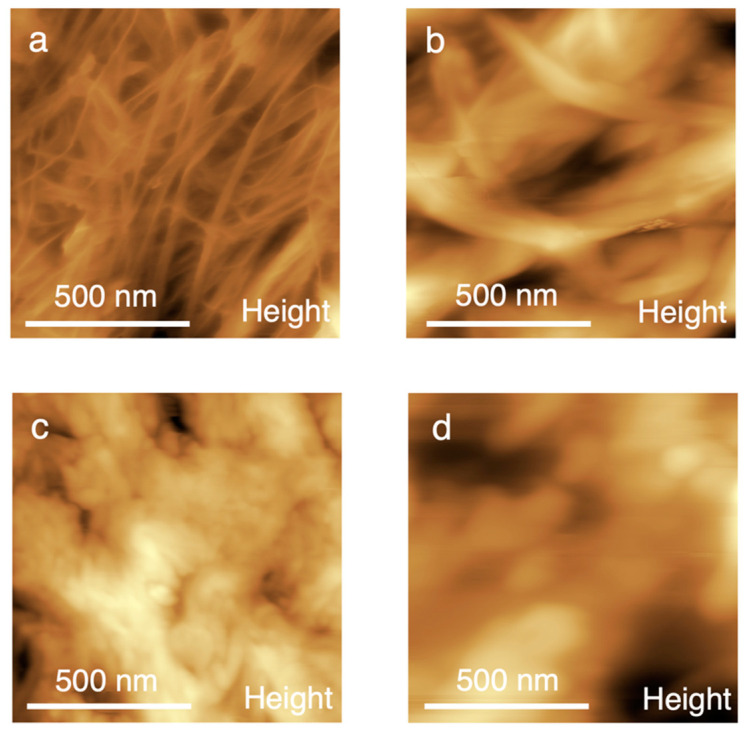
AFM images for M-CNF-0 (**a**), M-CNF-5 (**b**), M-CNF-12 (**c**), and M-CNF-30 (**d**).

In order to visualize the difference in the structure between prepared samples, an AFM study was performed on the 3D printed filaments after UV-curing. The topographies of the surfaces given in Figure 2 demonstrate that, for an anhydrous system, the individual M-CNFs are clearly visible and form the network (Figure 2a). A gradual increase in water content results in a growing degree of coagulation: Figure 2b for the M-CNF-5 sample demonstrates a network of nanofibers coagulating with each other, while, in Figure 2c,d, corresponding to M-CNF-12 and M-CNF-30, respectively, the nanofibrous structure is completely masked. In the case of the M-CNF-12 sample, the surface is rougher than in the case of the M-CNF-30 sample. This can be connected with increasing agglomeration as the water content rises. The larger sizes of the agglomerates and the larger pore diameters are also typical of M-CNF-30 gel. 

### 3.3. Rheological Properties of Inks 

**Figure 3:**
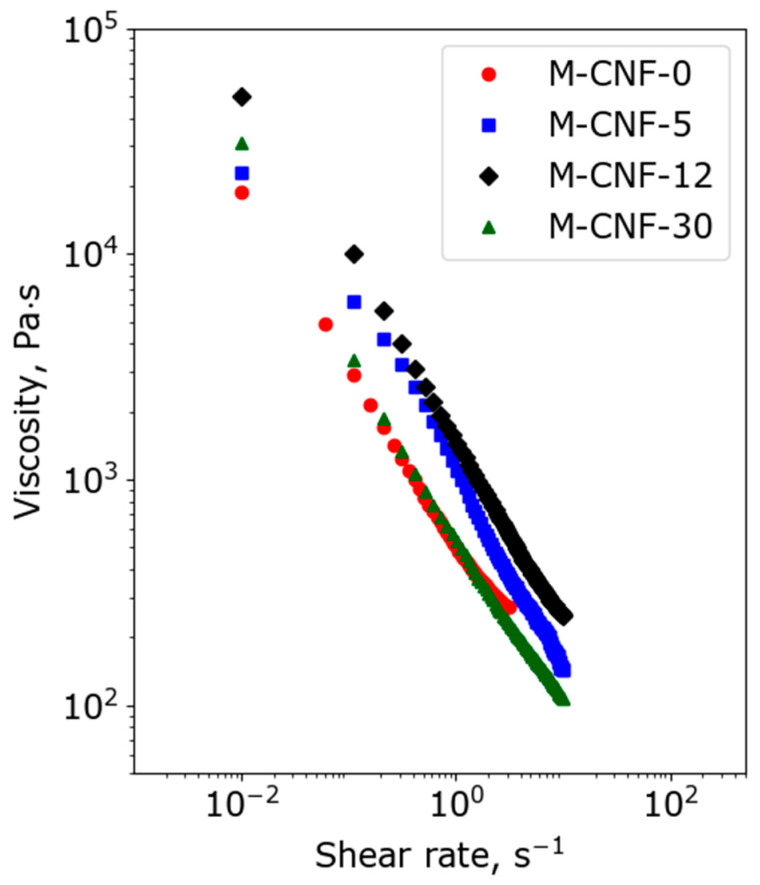
Viscosity curves of M-CNF samples in the DES and DES-water mixtures.

In order to determine the water influence on the rheological properties of the inks containing M-CNF dispersed in AA/ChCl with various water contents before 3D printing, flow curves in shear mode were measured, and the results are given in Figure 3. It can be seen that all of the investigated dispersions are non-Newtonian liquids with shear thinning behavior; a monotonous decrease in viscosity with increasing shear rate is observed. This is connected with the destruction of the cellulose nanofiber network and M-CNF orientation along the flow direction. The rheological properties of the studied dispersions make them suitable for 3D printing.

**Figure 4:**
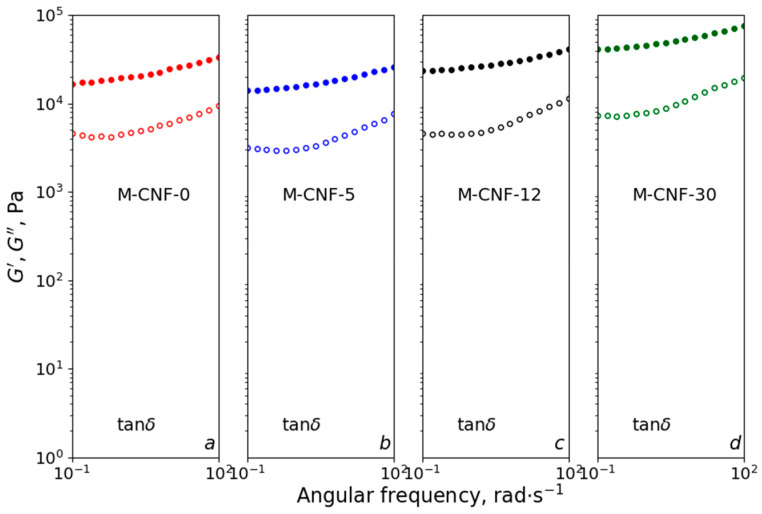
Storage (*G*′, filled symbols) and loss (*G*″, hollow symbols) moduli versus angular frequency for M-CNF dispersions with different water contents.

As can be seen in Figure 3, the viscosity values at a shear rate of 0.01 s^−1^ for M-CNF-0, M-CNF-5, M-CNF-12, and M-CNF-30 can be estimated as 18.8, 23.0, 49.7, and 31.1 kPa∙s, respectively. Thus, the maximal viscosity is observed for inks based on M-CNF-12, and this trend is also visible for other shear rates. This result can be explained as follows. The addition of water reduces the efficiency of the interactions of M-CNF with DES because of the hydration of polar ions in DES [47]. Thus, the stabilization of hydrophobic M-CNF in ink is reduced and the interactions between CNFs are enhanced. As a result, the viscosity increases due to the growing number of interactions between M-CNFs. The lower viscosity of M-CNF-30 ink in comparison with M-CNF-12 ink can be attributed to the more pronounced coagulation of M-CNF, which allows for the possibility of the rupture of a continuous M-CNF network in inks with the formation of coacervates. To verify this assumption, the yield strength for all inks was determined using the dependences of storage (*G’*) and loss (*G″*) moduli on shear stress (Figure S2) measured in dynamic mode. It was found that yield strength values for M-CNF-0, M-CNF-5, M-CNF-12, and M-CNF-30 were 541, 413, 790, and 453 Pa, respectively. The maximal value for M-CNF-12 ink confirms the assumption given above and demonstrates that the addition of 12 wt% of water leads to the formation of the strongest coagulation structures. The dependences of storage and loss moduli on angular frequency (see Figure 4) show that, for all samples, *G*′ > *G*″, which is typical for gels with solid-like behavior. Such gels can be considered to be structured dispersions (coagulation structures) [48]. It can be seen that the addition of 5 wt% of water to the anhydrous ink leads to a decrease in *G*′ and *G*″ values that can be attributed to the increasing ink mobility due to the intercalation of water molecules to the network of hydrogen bonds in DES. A further increase in the modulus for M-CNF-12 and M-CNF-30 inks gives additional evidence for the hypothesis of the formation of coagulation structures with enhanced interaction between modified CNFs due to the presence of water.

### 3.4. Mechanical Properties

The mechanical properties of the gels after UV-curing were measured using filaments obtained by 3D printing of the corresponding inks. It was supposed that closer contacts between M-CNFs in water-containing inks would result in more pronounced crosslinking of them via UV-induced radical polymerization of AA in DES and the TMSPM shell on the CNF surface. To check the conversion of acrylates, FTIR spectra of ink before and after UV-curing were obtained and are given in Figure S4. The acrylic acid bands at 1617, 1635, and 1725 cm^−1^ are clearly visible on the spectra before UV-curing [31]. On FTIR spectra of M-CNFs in polymerized DES, bands near 1617 and 1635 cm^−1^, corresponding to the double bond vibrations, are completely disappeared, which demonstrates the high conversion of the acrylic monomer into the polymerized DES.

**Figure 5:**
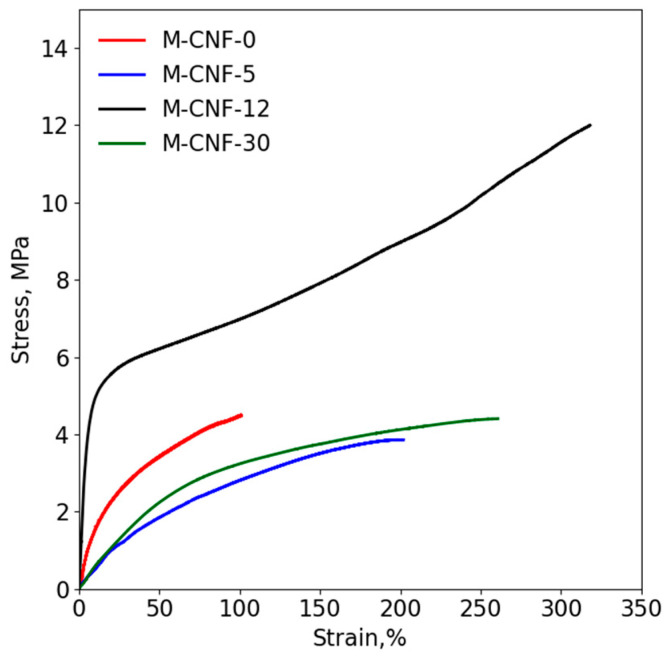
Stress–strain curves of the 3D-printed filaments, measured in the extension mode.

It was expected that the addition of water would improve the mechanical properties of the 3D printed samples. In order to exclude the plasticizing effect of water on polyacrylate, the water was removed from the samples after UV-curing via drying in a vacuum oven at 60 °C until the constant mass was reached before the mechanical measurements. The stress–strain curves given in Figure 5 demonstrate that all samples prepared with water have a higher elongation at break in comparison with sample printed in anhydrous conditions (see Table 1). This can be attributed to the formation of a continuous network of crosslinked M-CNFs. The highest strength of 11.9 ± 0.9 MPa is observed for the M-CNF-12 sample, which is two times higher than 5.5 ± 0.9 MPa for anhydrous filaments. In the case of lower (5 wt%) and higher (30 wt%) water content, the ultimate strength decreases to lower values than for initial anhydrous printed filament. The highest Young’s modulus of 103 ± 10 MPa is also observed for the M-CNF-12 sample, and the variation of its value with water content is the same as for the ultimate strength. It can be assumed that, at the lowest water content, the structure of the composite gel is loosened, with water in DES disrupting the network of hydrogen bonds between its components. At the same time, the coagulation of M-CNF-5 is not enough to form a strong network after UV-curing. Increasing the water content up to 12 wt% results in a rise in the number and density of entanglements of the network formed with M-CNF. This structure is fixed due to crosslinking, which leads to the stiffening and strengthening of the material. Further, increasing the water content higher than 12 wt% led to the dilution of DES, thus reducing the density of formed pDES and its mechanical properties. Additionally, when the water content increases up to 30 wt%, the excessive coagulation of M-CNFs and the formation of a non-uniform network with weaker connections between the agglomerates takes place. Also, the ionic network structure of DES at this water content is disrupted to a large extent. Concluding this discussion, it can be pointed out that the results of the mechanical measurements are in good agreement with the rheological investigations and confirm that the addition of 12 wt% of water facilitates the formation of the strongest coagulation structures due to the optimal balance between a degree of coagulation and the number of contacts between M-CNFs. 

It is interesting to compare the obtained characteristics with ones for other polymer gels reinforced with CNF reported in literature. In the work [49], the authors created a multifunctional conductive sensor based on polyvinyl alcohol reinforced with various CNF contents. Sample with 4% CNF had superior strength and elasticity in comparison with the pure hydrogel (for strength—2.88 MPa vs. 0.63 MPa; for Young’s modulus—0.63 MPa vs. 0.16 MPa). Das et al. [50] successfully increased, by three times, the strength and elasticity of the nanocomposite cellulose hydrogel reinforced with CNF. The researchers managed to achieve an increase in the mechanical characteristics of these systems by adding only 0.7 wt% of CNF (for the strength—16.27 MPa vs. 5.35 MPa; for the Young’s modulus—0.74 MPa vs. 0.23 MPa) in comparison with pure composite. In another work [51], the authors added 0.7 wt% of CNF to the pure poly (ethylene oxide)-poly(propylene oxide)-poly(ethylene oxide) block copolymer diacrylate, but, in this case, the strength and elastic modulus were increased only by 37% and 53% and reach 78 kPa and 148 kPa, respectively. Compared to the results obtained in our work for the M-CNF-12 sample, the earlier reported systems usually demonstrated comparable or even lower mechanical properties. This confirms that our systems are capable of being used as a prospective functional material.

It is also interesting to note that the shape of the stress–strain curve demonstrates that the mechanical behavior of the M-CNF-0 sample is common for thermoplastic polymers, while that of the M-CNF-5 and the M-CNF-30 is common for elastomeric ones. The mechanical behaviors of M-CNF-12 are typical of thermoelastomeric polymers. This allows one to suggest the existence of two sub-networks, both of them significantly affecting the mechanical behavior. The first is the chemically crosslinked network of M-CNFs, while the second is the physically crosslinked network of pDES based on ionic interactions between choline chloride and polyacrylic acid.

### 3.5. Dynamic Mechanical Analysis 

**Figure 6:**
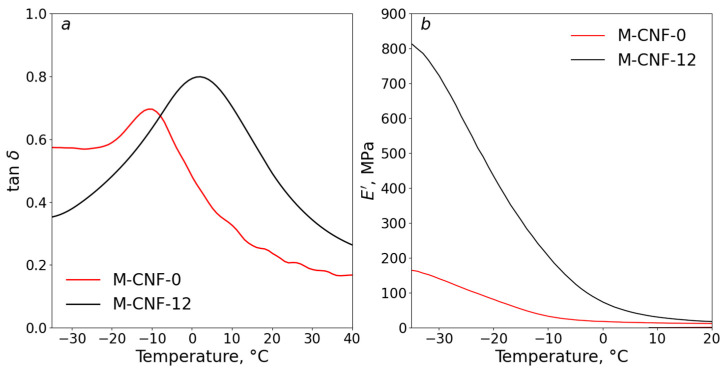
Dependences of the loss tangent tan *δ* (**a**) and the real component of elastic modulus *E’* (**b**) for the M-CNF-0 and M-CNF-12 inks after 3D printing and UV-curing.

Relaxation transitions were studied using DMA. In the experiments, the printed and subsequently UV-cured formulations of M-CNF-0 and M-CNF-12 gels were selected as a reference and as a sample with the highest strength provided by a coagulation structure. The loss tangent (tan *δ*) and dynamic modulus of elasticity (*E*′) were measured in the temperature range from −35 °C to 50 °C and are given in Figure 6a and b, respectively. 

On the curves *E’*(*T*), the beginning of a decrease in the value of the storage modulus during the transition of the material from a glassy state to an elastic one corresponds to the maximum dynamic stiffness of the samples (Table 2). The value of *E*’ is higher for the M-CNF-12 sample in comparison with the M-CNF-0 sample (Figure 6b). This can be attributed to the higher stiffness of the mesh nodes due to the formation of a coagulation structure and was anticipated on the basis of the results of the mechanical measurements. The subsequent fixation of the structure with UV-induced polymerization allows it to effectively accumulate mechanical energy while deforming and returning to the initial shape when the load is removed.

The value of the glass transition temperature (*T_g_*) that is attributed to the pDES phase of the obtained gels [52] can be estimated from the maximums in the dependencies of tan *δ* on *T* that are given in Figure 6a. The segmental mobility in the samples is affected by the coagulation structure formed in the presence of 12% water. It limits the mobility of pDES macromolecules, since polyacrylic acid chains are covalently bound to TMSPM on the CNF surface, which contributes to an increase in *T*_g_ from −11 °C for M-CNF-0 to 3 °C for M-CNF-12.

### 3.6. Shape Memory Properties

**Figure 7:**
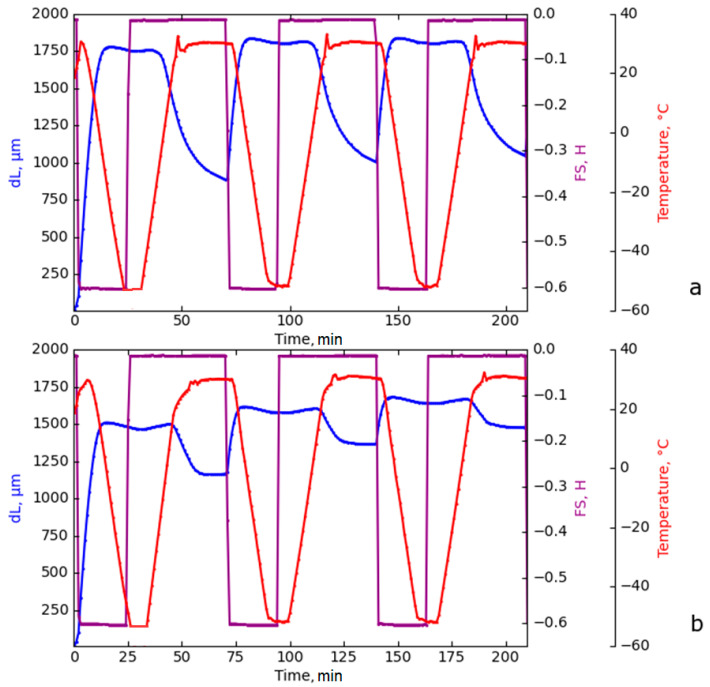
Cyclic thermomechanical tests of the samples: M-CNF-0 (**a**), M-CNF-12 (**b**) in the DMA mode at FS = 0.6H.

The shape memory properties were studied for the 3D printed models of the composite gels demonstrating the highest deformation-strength characteristics (Table 1), namely M-CNF-0 and M-CNF-12. The tests were carried out in dynamic mode using a DMA setup and in static mode with the help of a device for the uniaxial orientation of the films placed in a climatic chamber. According to the measurement in dynamic mode, both samples demonstrate high values of the shape fixation coefficient (*R_f_*), about 99% (Table 3). The addition of water to the hydrogel composition during 3D printing of the samples practically does not affect the *R_f_* value, but significantly reduces the shape recovery coefficient (*R_r_*). It should be noted that, in the process of thermomechanical cycling, the *R_r_* value increased both for the samples formed without water (Figure 7a) and for the sample M-CNF-12 (Figure 7b). As can be seen from Table 3, the recovery parameters *R****_r_*** increase with each thermomechanical cycle; therefore, the effect of shape memory training is observed [53]. After the third cycle, the recovery coefficient of the anhydrous sample M-CNF-0 was 93.8%, while, for the M-CNF-12, this parameter did not exceed 63.2% (Table 3). The values of *R_f_* and *R_r_* obtained using the DMA method for the printed models of the composite gels are comparable to those reported in the literature for hydrogels based on thermoplastic polyurethanes and microcrystalline cellulose. Cai et al. [54] obtained composite gels with a thermally induced SME based on a polyurethane matrix (Desmopan DP 2795A), rhodamine B, and chemically modified microcrystalline cellulose with 1-allyl-3-methylimidazolium chloride, which had a high degree of temporary fixation (98.8%) and recovery to initial forms (91.4%).

The peculiarity of measuring the shape memory effect by using a DMA machine is the low relative elongation of the samples under load, which is within 10% of their original length. This small deformation is within one order of magnitude of the residual deformation that was observed after the cyclic thermomechanical tests on the 3D printed models in static mode, where the elongation was 60% (Table 4). The deformation of the samples at temperatures above *T_g_* of pDES leads to a conformational change in the M-CNF network and the accumulation of mechanical stresses in it. The subsequent freezing at *T* < *T_g_* of the pDES phase fixes the shape of the material, and the mechanical energy remains stored within the M-CNF network. At small deformations, the stretched sample of the 3D printed model in the shape of a dog bone below the *T_g_* is fixed better, since the rigidity of the frozen ion gel is sufficient to resist the stress caused by stretching the M-CNF network. At the same time, the energy accumulated at small deformations is not enough to induce the reorganization of the network of hydrogen bonds in the pDES phase, which makes it difficult for the 3D printed model to restore its original size. Moreover, according to the DMA data discussed above, the mobility of the DES chains in the M-CNF-12 model is lower than for the M-CNF-0 sample (it needs more energy for reorganization). Thus, at low deformations during the DMA experiment, the rigidity of the crosslinked CNF network is not enough to recover its initial shape in the case of the M-CNF-12 sample.

In the static mode, when the M-CNF-0 and M-CNF-12 3D printed models in the shape of dog bones were elongated by 60%, the removal of the load after cooling was accompanied by the creep of the composite gels, bringing the *R_f_* values down to 80.0 and 16.7%, respectively (Table 4). For the M-CNF-12 sample, the poor fixation of the temporal shape may be due to the fact that the DES phase, even in the glassy state, is not able to restrain the rigid coagulation structure of M-CNF in the deformed state. While deformed, the chemically crosslinked network tends to return to its original state and minimize the mechanical stress brought about by uniaxial tension due to the energy accumulated within it. The subsequent heating of the M-CNF-12 model to 50 °C contributed to further restoration of its original dimensions, while the residual deformation of the sample was 6.7% (Figure 1). In the case of M-CNF-0, the energy of the network of reinforcing filler was insufficient to compress the ion gel in the glassy state, which contributed to the effective fixation of the temporary shape by the sample. Unfreezing the mobility of the DES chains above the temperature of its relaxation transition led to the removal of mechanical stresses in the reinforced M-CNF network and, accordingly, the restoration of the initial dimensions of the sample.

## 4. Conclusions

Composite hydrogels with different water contents based on modified cellulose nano-fibers and pDES, which is a mixture of acrylic acid and choline chloride, have been obtained. Investigation of the rheological properties demonstrated that the increase in water content in the samples resulted in nonmonotonic dependence of the viscosity and mechanical properties of the prepared materials. It was proposed that adding water reduces the efficiency of the interactions of M-CNF with DES due to the solvation of polar components and ions in DES. As a result, the interactions between M-CNFs are enhanced. Thus, the viscosity and yield strength reach maximum values for the system with optimal balance in reducing the DES viscosity and the formation of the coagulation structure with the M-CNF dispersed in it. In the case of the reported system, the optimal balance is reached at 12 wt% water content. At higher water content, the formation of aggregates with weak connections, decreasing the viscosity of the ink, can be proposed. Consequently, using inks with optimal water content, the samples with the best mechanical properties can be 3D printed. After UV-curing, the strength of sample prepared at optimal conditions is 11.9 MPa, which is two times higher than 5.5 MPa for anhydrous system. 

It has been shown that, due to the double network structure, consisting of a chemically crosslinked M-CNF sub-network and a physically crosslinked pDES sub-network, the 3D printed composite gels exhibit the shape memory effect. The relaxation transition of the pDES from the glassy to the rubber state is responsible for the fixation of the temporal shape in them, and the restoration of the initial dimensions is provided by a chemically crosslinked M-CNF sub-network. The best shape memory properties were found for the sample prepared without water: shape fixation and shape recovery coefficients reached 80.0 and 95.8%, respectively, in static mode and 98.8 and 93.8%, respectively, after three cycles of training in dynamic mode. At the same time, when the chemically crosslinked sub-network is stiffened due to the formation of a coagulation structure in the presence of water, the shape memory properties deteriorate due to the manifestation of the creep effect. Below *T_g_*, the pDES phase is unable to keep the reinforced M-CNF network in a deformed state. Therefore, when developing actuators and soft robots based on such gels, it is important to keep a balance between the rigidity of the reinforcing frame and the energy of the reorientation of interactions in the pDES component.

## Figures and Tables

**Table 1:** Mechanical characteristics of the samples in extension mode.

Samples	Young’s Modulus, MPa	Tensile Strength, MPa	Elongation at Break, %
M-CNF-0	23 ± 2	5.5 ± 0.9	100 ± 5
M-CNF-5	5.3 ± 0.7	3.4 ± 0.4	202 ± 19
M-CNF-12	103 ± 10	11.9 ± 0.9	300 ± 60
M-CNF-30	11.3 ± 3.8	4.4 ± 0.9	224 ± 41

**Table 2:** Dynamic mechanical characteristics of the 3D printed models for composite gels with different water contents.

Sample	*E′*_max_, MPa	*T_g_*, °C
M-CNF-0	173	−11
M-CNF-12	829	3

**Table 3:** Shape memory properties of the 3D printed models in the shape of a dog bone, measured in the DMA mode.

Samples	No. Cycle	*ε*_0_, μm	*ε_m_*, μm	*ε*, μm	*ε_re_*_c_, μm	*R_f_*,%	*R_r_*,%
M-CNF-0	1	45	1779	1759	887	98.9	50.9
	2	887	1836	1815	1011	98.9	86.7
	3	1011	1839	1817	1062	98.8	93.8
M-CNF-12	1	30	1511	1500	1163	99.3	22.9
	2	1163	1617	1605	1366	99.3	54.1
	3	1366	1681	1670	1478	99.3	63.2

**Table 4:** Shape memory properties of the 3D printed M-CNFs samples, measured in static mode.

Samples	*L_i_*, mm	*L_d_*, mm	*L_u_*, mm	*L_r_*, mm	*R_fs_*,%	*R_rs_*,%
M-CNF-0	50	80	74	51	80.0	95.8
M-CNF-12	50	80	55	52	16.7	60.0

## Data Availability

Data are available within the article or its supplementary materials.

## Supplementary Materials

The following supporting information can be downloaded at: https://www.mdpi.com/article/10.3390/polym15173547/s1, Figure S1: Accumulation (G′, filled symbols) and loss (G″, hollow symbols) moduli versus deformation for M-CNF dispersions; Figure S2: Storage (G′, filled symbols) and loss (G″, hollow symbols) moduli versus shear stress for dispersions of M-CNF; Figure S3: The flow diagrams of the nozzle during 3D printing of filaments (a) and dog bone shapes (b); Figure S4: FTIR spectra of the M-CNF dispersions in DES before and after UV-curing; Figure S5. Scheme of obtaining 3D printed models in the shape of dog bones.

## Abbreviations

AA	Acrylic acid
AFM	Atomic force microscopy
ChCl	Choline chloride
CNF	Cellulose nanofibers
DES	Deep eutectic solvent
DMA	Dynamic mechanical analysis
DN	Double network
FTIR	Fourier transform infrared spectroscopy
GelMA	Methacrylated gelatin
LVE	Linear viscoelastic region
M-CNF-0	Sample with anhydrous 4wt% TMSPM-modified cellulose nanofibers
M-CNF-5	Sample with 4wt% TMSPM-modified cellulose nanofibers and 5 wt% of water
M-CNF-12	Sample with 4wt% TMSPM-modified cellulose nanofibers and 12 wt% of water
M-CNF-30	Sample with 4wt% TMSPM-modified cellulose nanofibers and 30 wt% of water
pDES	Polymerized deep eutectic solvent
PVA	Polyvinyl alcohol
SMP	Shape memory properties
TMSPM	3-(trimethoxysilyl) propyl methacrylate

## References

[1] Lionetto F., Sannino A., Mensitieri G., Maffezzoli A. (2003). Evaluation of the Degree of Cross-Linking of Cellulose-Based Superabsorbent Hydrogels: A Comparison between Different Techniques. Macromol. Symp..

[2] Zhao B., Jiang H., Lin Z., Xu S., Xie J., Zhang A. (2019). Preparation of Acrylamide/Acrylic Acid Cellulose Hydrogels for the Adsorption of Heavy Metal Ions. Carbohydr. Polym..

[3] Ganesan M., Juvekar V.A. (2020). Reduction Self-Assembly of Three-Dimensional Graphene Hydrogels: Implication as Adsorbents. ACS Appl. Nano Mater..

[4] Oh M.J., Yoo P.J. (2020). Graphene-Based 3D Lightweight Cellular Structures: Synthesis and Applications. Korean J. Chem. Eng..

[5] Areyano M., Valois E., Sanchez Carvajal I., Rajkovic I., Wonderly W.R., Kossa A., McMeeking R.M., Waite J.H. (2022). Viscoelastic Analysis of Mussel Threads Reveals Energy Dissipative Mechanisms. J. R. Soc. Interface.

[6] Waite J.H., Vaccaro E., Sun C., Lucas J.M. (2002). Elastomeric Gradients: A Hedge against Stress Concentration in Marine Holdfasts?. Philos. Trans. R. Soc. B Biol. Sci..

[7] Qin Z., Yu X., Wu H., Yang L., Lv H., Yang X. (2020). Injectable and Cytocompatible Dual Cross-Linking Hydrogels with Enhanced Mechanical Strength and Stability. ACS Biomater. Sci. Eng..

[8] Takashima Y., Hatanaka S., Otsubo M., Nakahata M., Kakuta T., Hashidzume A., Yamaguchi H., Harada A. (2012). Expansion-Contraction of Photoresponsive Artificial Muscle Regulated by Host-Guest Interactions. Nat. Commun..

[9] Xia L.W., Xie R., Ju X.J., Wang W., Chen Q., Chu L.Y. (2013). Nano-Structured Smart Hydrogels with Rapid Response and High Elasticity. Nat. Commun..

[10] Bierbrauer K.L., Alasino R.V., Barclay F.E., Belotti E.M., Ortega H.H., Beltramo D.M. (2021). Biocompatible Hydrogel for Intra-Articular Implantation Comprising Cationic and Anionic Polymers of Natural Origin: In Vivo Evaluation in a Rabbit Model. Polymers.

[11] Zhao H., Liu M., Zhang Y., Yin J., Pei R. (2020). Nanocomposite Hydrogels for Tissue Engineering Applications. Nanoscale.

[12] Zhang L., Li K., Xiao W., Zheng L., Xiao Y., Fan H., Zhang X. (2011). Preparation of Collagen-Chondroitin Sulfate-Hyaluronic Acid Hybrid Hydrogel Scaffolds and Cell Compatibility in Vitro. Carbohydr. Polym..

[13] Spicer C.D. (2020). Hydrogel Scaffolds for Tissue Engineering: The Importance of Polymer Choice. Polym. Chem..

[14] Choi S.M., Rao K.M., Zo S.M., Shin E.J., Han S.S. (2022). Bacterial Cellulose and Its Applications. Polymers.

[15] Almeida A.P.C., Saraiva J.N., Cavaco G., Portela R.P., Leal C.R., Sobral R.G., Almeida P.L. (2022). Crosslinked Bacterial Cellulose Hydrogels for Biomedical Applications. Eur. Polym. J..

[16] Lei Z., Wu P. (2019). A Highly Transparent and Ultra-Stretchable Conductor with Stable Conductivity during Large Deformation. Nat. Commun..

[17] Lee Y., Song W.J., Sun J.Y. (2020). Hydrogel Soft Robotics. Mater. Today Phys..

[18] Sun X., Yao F., Li J. (2020). Nanocomposite Hydrogel-Based Strain and Pressure Sensors: A Review. J. Mater. Chem. A.

[19] Naficy S., Kawakami S., Sadegholvaad S., Wakisaka M., Spinks G.M. (2013). Mechanical Properties of Interpenetrating Polymer Network Hydrogels Based on Hybrid Ionically and Covalently Crosslinked Networks. J. Appl. Polym. Sci..

[20] Xu P., Shang Z., Yao M., Ke Z., Li X., Liu P. (2022). Molecular Insights on the Mechanical Properties of Double-Network Hydrogels Reinforced by Covalently Compositing with Silica-Nanoparticles. J. Mol. Liq..

[21] Zeng Y., Yang J., Huang K., Lee Z., Lee X. (2001). A Comparison of Biomechanical Properties between Human and Porcine Cornea. J. Biomech..

[22] Guldberg R.E., Duty A.O. (2006). Design Parameters for Engineering Bone Regeneration.

[23] Zhang X.N., Zheng Q., Wu Z.L. (2022). Recent Advances in 3D Printing of Tough Hydrogels: A Review. Compos. Part B Eng..

[24] Huang Y., Qian S., Zhou J., Chen W., Liu T., Yang S., Long S., Li X. (2023). Achieving Swollen yet Strengthened Hydrogels by Reorganizing Multiphase Network Structure. Adv. Funct. Mater..

[25] Li B., Wu C., Han Y., Ma X., Luo Z. (2021). Preparation of Poly(Acrylic Acid) Grafted Reduced Graphene Oxide/Polyacrylamide Composite Hydrogels with Good Electronic and Mechanical Properties by in-Situ Polymerization. J. Macromol. Sci. Part B Phys..

[26] Yang C., Liu Z., Chen C., Shi K., Zhang L., Ju X.J., Wang W., Xie R., Chu L.Y. (2017). Reduced Graphene Oxide-Containing Smart Hydrogels with Excellent Electro-Response and Mechanical Properties for Soft Actuators. ACS Appl. Mater. Interfaces.

[27] Huang Y., Zhang M., Ruan W. (2014). High-Water-Content Graphene Oxide/Polyvinyl Alcohol Hydrogel with Excellent Mechanical Properties. J. Mater. Chem. A.

[28] Park D., Kim J.W., Shin K., Kim J.W. (2021). Bacterial Cellulose Nanofibrils-Reinforced Composite Hydrogels for Mechanical Compression-Responsive on-Demand Drug Release. Carbohydr. Polym..

[29] Yuan N., Xu L., Zhang L., Ye H., Zhao J., Liu Z., Rong J. (2016). Superior Hybrid Hydrogels of Polyacrylamide Enhanced by Bacterial Cellulose Nanofiber Clusters. Mater. Sci. Eng. C.

[30] Smirnov M.A., Fedotova V.S., Sokolova M.P., Nikolaeva A.L., Elokhovsky V.Y., Karttunen M. (2021). Polymerizable Choline-and Imidazolium-Based Ionic Liquids Reinforced with Bacterial Cellulose for 3D-Printing. Polymers.

[31] Vorobiov V.K., Sokolova M.P., Bobrova N.V., Elokhovsky V.Y., Smirnov M.A. (2022). Rheological Properties and 3D-Printability of Cellulose Nanocrystals/Deep Eutectic Solvent Electroactive Ion Gels. Carbohydr. Polym..

[32] Hu D., Zeng M., Sun Y., Yuan J., Wei Y. (2021). Cellulose—Based Hydrogels Regulated by Supramolecular Chemistry. SusMat.

[33] Liu T., Chen W., Li K., Long S., Li X., Huang Y. (2023). Toughening Weak Polyampholyte Hydrogels with Weak Chain Entanglements via a Secondary Equilibrium Approach. Polymers.

[34] Gong J.P., Katsuyama Y., Kurokawa T., Osada Y. (2003). Double-Network Hydrogels with Extremely High Mechanical Strength. Adv. Mater..

[35] Zhuang Y., Yu F., Chen H., Zheng J., Ma J., Chen J. (2016). Alginate/Graphene Double-Network Nanocomposite Hydrogel Beads with Low-Swelling, Enhanced Mechanical Properties, and Enhanced Adsorption Capacity. J. Mater. Chem. A.

[36] Jing Z., Xu A., Liang Y.Q., Zhang Z., Yu C., Hong P., Li Y. (2019). Biodegradable Poly(Acrylic Acid-Co-Acrylamide)/ Poly(Vinyl Alcohol) Double Network Hydrogels with Tunable Mechanics and High Self-Healing Performance. Polymers.

[37] Hagiwara Y., Putra A., Kakugo A., Furukawa H., Gong J.P. (2010). Ligament-like Tough Double-Network Hydrogel Based on Bacterial Cellulose. Cellulose.

[38] Buyanov A.L., Gofman I.V., Saprykina N.N. (2019). High-Strength Cellulose–Polyacrylamide Hydrogels: Mechanical Behavior and Structure Depending on the Type of Cellulose. J. Mech. Behav. Biomed. Mater..

[39] Visser J., Melchels F.P.W., Jeon J.E., Van Bussel E.M., Kimpton L.S., Byrne H.M., Dhert W.J.A., Dalton P.D., Hutmacher D.W., Malda J. (2015). Reinforcement of Hydrogels Using Three-Dimensionally Printed Microfibres. Nat. Commun..

[40] Gao F., Xu Z., Liang Q., Li H., Peng L., Wu M., Zhao X., Cui X., Ruan C., Liu W. (2019). Osteochondral Regeneration with 3D-Printed Biodegradable High-Strength Supramolecular Polymer Reinforced-Gelatin Hydrogel Scaffolds. Adv. Sci..

[41] Prosvirnina A.P., Bugrov A.N., Dobrodumov A.V., Vlasova E.N., Fedotova V.S., Nikolaeva A.L., Vorobiov V.K., Sokolova M.P., Smirnov M.A. (2022). Bacterial Cellulose Nanofibers Modification with 3-(Trimethoxysilyl)Propyl Methacrylate as a Crosslinking and Reinforcing Agent for 3D Printable UV-Curable Inks. J. Mater. Sci..

[42] Zhang Y., Jiang L., Zhang H., Li Q., Ma N., Zhang X. (2023). High-Strength Double-Network Conductive Hydrogels Based. Molecules.

[43] Rehbinder P.A. (1965). Formation of Structures in Disperse Systems. Pure Appl. Chem..

[44] Uriev N.B. (2004). Physicochemical Dynamics of Disperse Systems. Usp. Khim..

[45] Sonntag H., Strenge K., Vincent B. (1987). Coagulation Kinetics and Structure Formation.

[46] Xu J., Song J. (2011). Thermal Responsive Shape Memory Polymers for Biomedical Applications.

[47] Fedotova V.S., Sokolova M.P., Vorobiov V.K., Sivtsov E.V., Lukasheva N.V., Smirnov M.A. (2023). Water Influence on the Physico-Chemical Properties and 3D Printability of Choline Acrylate—Bacterial Cellulose Inks. Polymers.

[48] Mezger T.G. (2006). The Rheology Handbook: For Users of Rotational and Oscillatory Rheometers.

[49] Li M., Chen D., Sun X., Xu Z., Yang Y., Song Y., Jiang F. (2022). An Environmentally Tolerant, Highly Stable, Cellulose Nanofiber-Reinforced, Conductive Hydrogel Multifunctional Sensor. Carbohydr. Polym..

[50] Das D., Bhattacharjee S., Bhaladhare S. (2023). Preparation of Cellulose Hydrogels and Hydrogel Nanocomposites Reinforced by Crystalline Cellulose Nanofibers (CNFs) as a Water Reservoir for Agriculture Use. ACS Appl. Polym. Mater..

[51] Jeencham R., Tawonsawatruk T., Numpaisal P.O., Ruksakulpiwat Y. (2023). Reinforcement of Injectable Hydrogel for Meniscus Tissue Engineering by Using Cellulose Nanofiber from Cassava Pulp. Polymers.

[52] Bednarz S., Fluder M., Galica M., Bogdal D., Maciejaszek I. (2014). Synthesis of Hydrogels by Polymerization of Itaconic Acid-Choline Chloride Deep Eutectic Solvent. J. Appl. Polym. Sci..

[53] Didenko A.L., Kamalov A.M., Smirnova V.E., Vaganov G.V., Popova E.N., Kuznetcov D.A., Svetlichnyi V.M., Yudin V.E., Kudryavtsev V.V. (2022). Multiblock (Segmented) Shape Memory Copolymers Containing Polyurethane and Rigid-Chain Polyimide Blocks. Russ. Chem. Bull..

[54] Cai C., Wei Z., Wang X., Mei C., Fu Y., Zhong W.H. (2018). Novel Double-Networked Polyurethane Composites with Multi-Stimuli Responsive Functionalities. J. Mater. Chem. A.

