# Supplementary material for: Three-Dimensional Printed Shape Memory Gels Based on a Structured Disperse System with Hydrophobic Cellulose Nanofibers
Source: Polymers (Basel). 2023 Aug 26;15(17):3547. doi: 10.3390/polym15173547 (PMC10490119; doi:10.3390/polym15173547)
Supplement: Supplementary file 1 [file polymers-15-03547-s001.zip › polymers-2554715-supplementary.pdf]

Article

Three-Dimensional Printed Shape Memory Gels Based on a Structured Disperse System with Hydrophobic Cellulose Nanofibers

Angelina P. Prosvirnina [1], Alexander N. Bugrov [1,2,*], Natalya V. Bobrova [1], Eugene V. Sivtsov [1,3], Alexandra L. Nikolaeva [1], Almaz M. Kamalov [1], Maria P. Sokolova [1,*] and Michael A. Smirnov [1,*]

[1] Institute of Macromolecular Compounds, Russian Academy of Sciences, Bolshoy Pr. 31, Saint Petersburg 199004, Russia
[2] Department of Physical Chemistry, Saint Petersburg Electrotechnical University (ETU "LETI"), ul. Professora Popova 5, Saint Petersburg 197022, Russia
[3] Saint Petersburg State Institute of Technology, Moskovsky Pr. 24-26/49, Saint Petersburg 190013, Russia
* Correspondence: bugrov.an@mail.ru (A.N.B.); pmarip@mail.ru (M.P.S.); smirnov_michael@mail.ru (M.A.S.)

1. **Experimental part.**

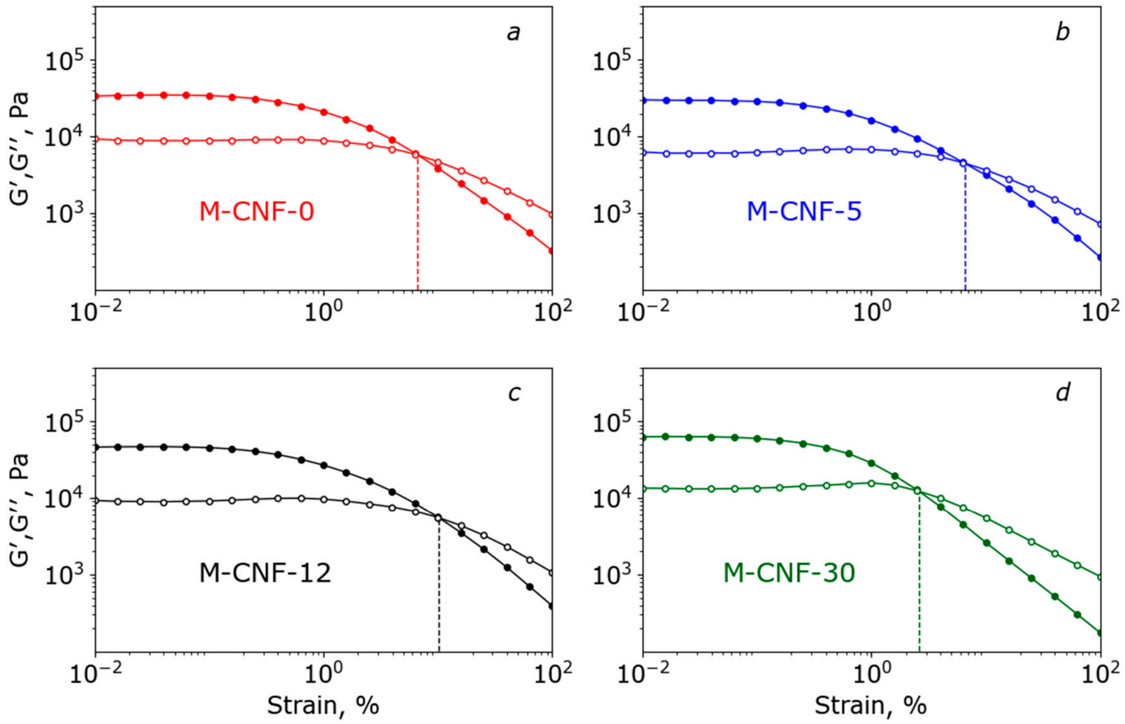

Figure S1. Accumulation (*G′*, filled symbols) and loss (*G′′*, hollow symbols) moduli versus deformation for M-CNFs dispersions with different water content.

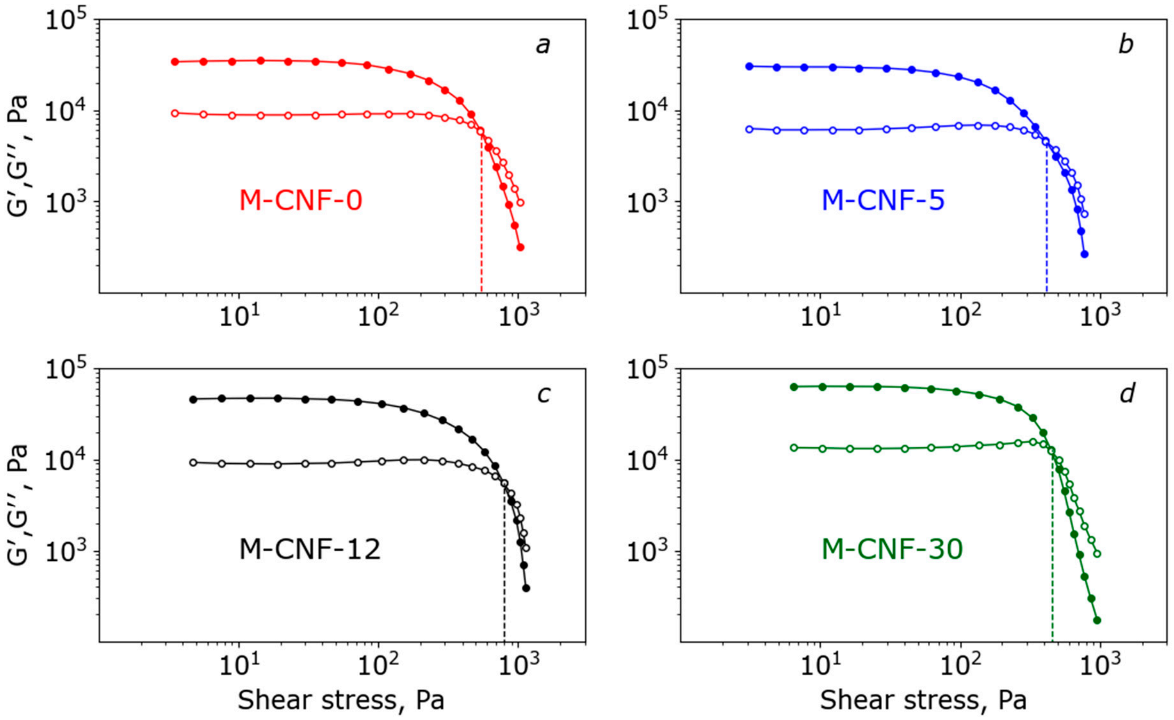

Figure S2. Storage (G', filled symbols) and loss (G'', hollow symbols) moduli versus shear stress for M-CNFs dispersions

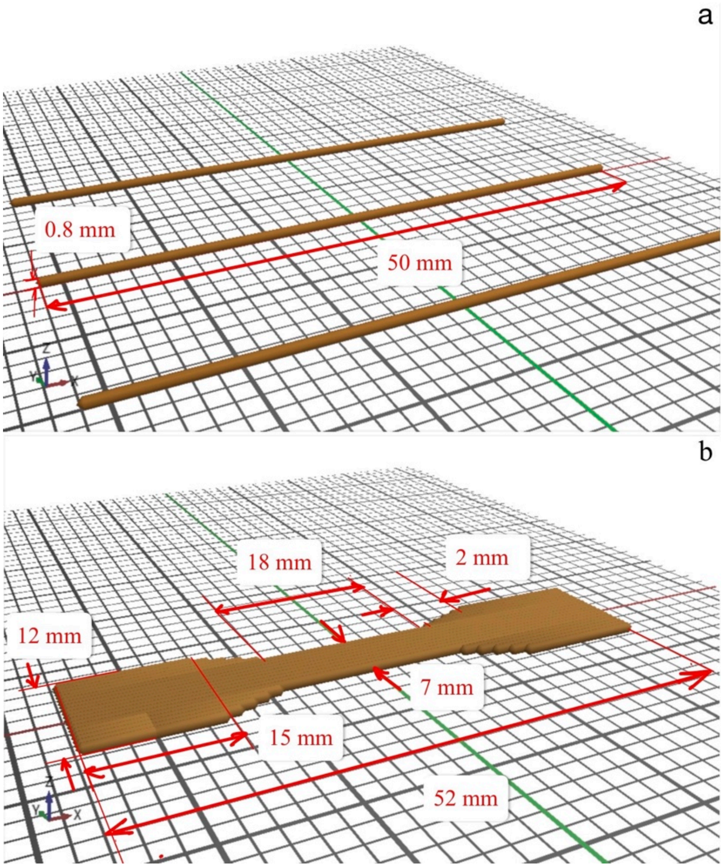

Figure S3. The flow diagrams of the nozzle during 3D printing of filaments (a) and dogbone shapes (b).

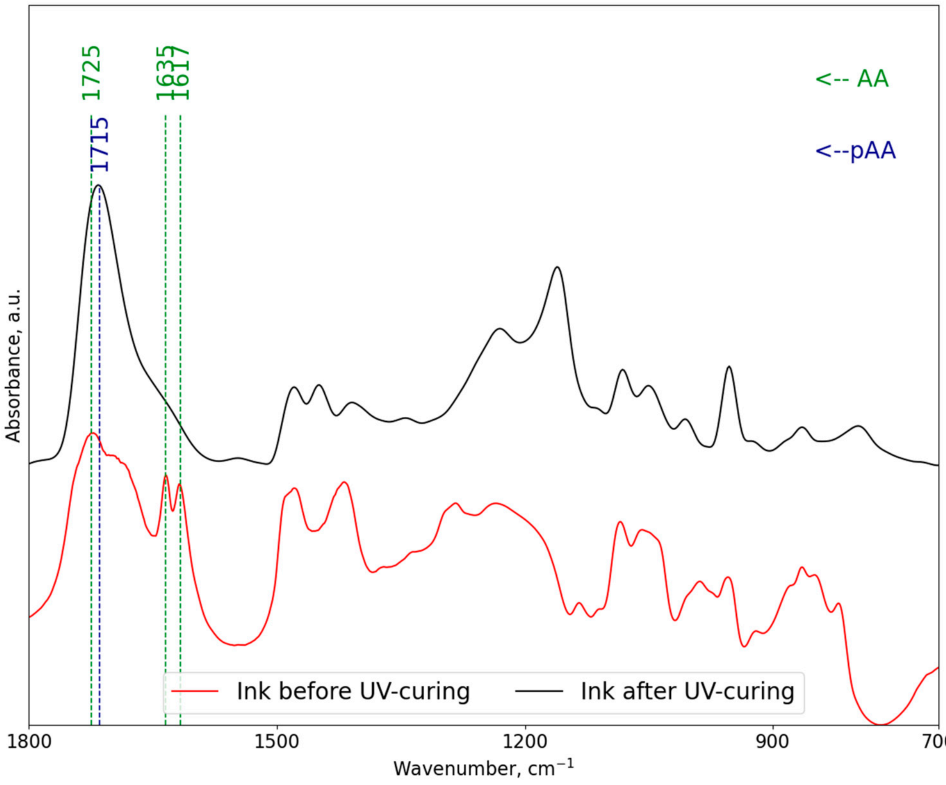

Figure S4. FTIR spectra of the M-CNF dispersions in DES before and after UV-curing. AA and pAA denotes acrylic acid and polyacrylic acid, respectively.

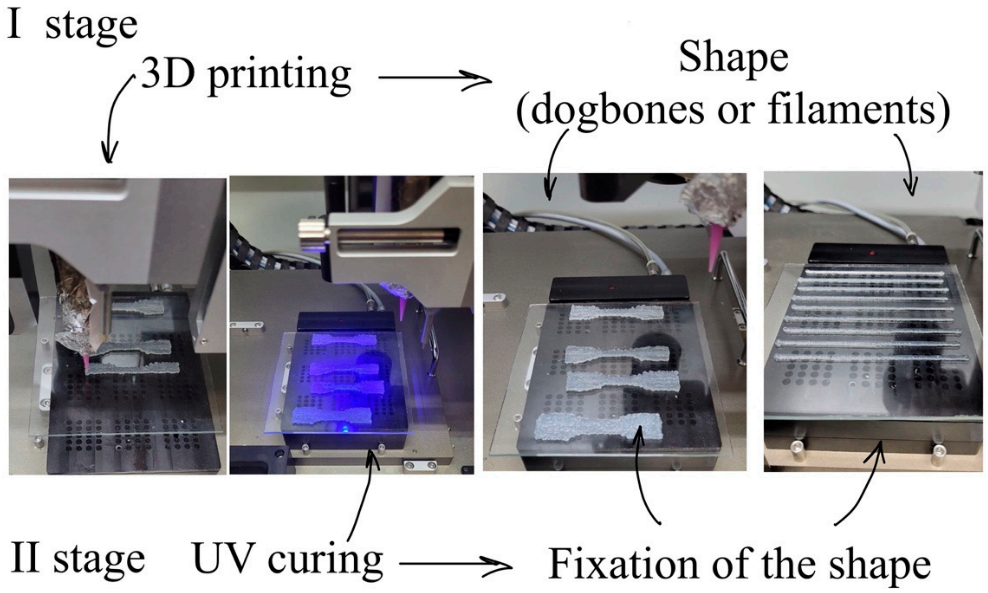

Figure S5. Scheme of obtaining 3D printed models in the shape of dogbones.
